# Fibrin clot properties independently predict adverse clinical outcome following acute coronary syndrome: a PLATO substudy

**DOI:** 10.1093/eurheartj/ehy013

**Published:** 2018-01-29

**Authors:** Wael Sumaya, Lars Wallentin, Stefan K James, Agneta Siegbahn, Katja Gabrysch, Maria Bertilsson, Anders Himmelmann, Ramzi A Ajjan, Robert F Storey

**Affiliations:** 1 Department of Infection, Immunity and Cardiovascular Disease, University of Sheffield, Beech Hill Road, Sheffield, S10 2RX, UK; 2 Department of Medical Sciences, Cardiology, Uppsala University, Uppsala, Sweden; 3 Uppsala Clinical Research Center, Uppsala University, Dag Hammarskjölds väg 38, SE-752 37 Uppsala, Sweden; 4 Department of Medical Sciences, Clinical Chemistry, Uppsala University, Uppsala, Sweden; 5 AstraZeneca Research and Development, Gothenburg, Sweden; 6 Leeds Institute of Cardiovascular and Metabolic Medicine, University of Leeds, Leeds, UK

**Keywords:** Acute coronary syndrome, Fibrin clot, Lysis time, Biomarker

## Abstract

**Aims:**

To determine whether fibrin clot properties are associated with clinical outcomes following acute coronary syndrome (ACS).

**Methods and results:**

Plasma samples were collected at hospital discharge from 4354 ACS patients randomized to clopidogrel or ticagrelor in the PLATelet inhibition and patient Outcomes (PLATO) trial. A validated turbidimetric assay was employed to study plasma clot lysis time and maximum turbidity (a measure of clot density). One-year rates of cardiovascular (CV) death, spontaneous myocardial infarction (MI) and PLATO-defined major bleeding events were assessed after sample collection. Hazard ratios (HRs) were estimated using Cox proportional hazards models. After adjusting for CV risk factors, each 50% increase in lysis time was associated with CV death/spontaneous MI [HR 1.17, 95% confidence interval (CI) 1.05–1.31; *P* < 0.01] and CV death alone (HR 1.36, 95% CI 1.17–1.59; *P* < 0.001). Similarly, each 50% increase in maximum turbidity was associated with increased risk of CV death (HR 1.24, 95% CI 1.03–1.50; *P* = 0.024). After adjustment for other prognostic biomarkers (leukocyte count, high-sensitivity C-reactive protein, high-sensitivity troponin T, cystatin C, N-terminal pro B-type natriuretic peptide, and growth differentiation factor-15), the association with CV death remained significant for lysis time (HR 1.2, 95% CI 1.01–1.42; *P* = 0.042) but not for maximum turbidity. These associations were consistent regardless of randomized antiplatelet treatment (all interaction *P* > 0.05). Neither lysis time nor maximum turbidity was associated with major bleeding events.

**Conclusion:**

Fibrin clots that are resistant to lysis independently predict adverse outcome in ACS patients. Novel therapies targeting fibrin clot properties might be a new avenue for improving prognosis in patients with ACS.

**Figure:**
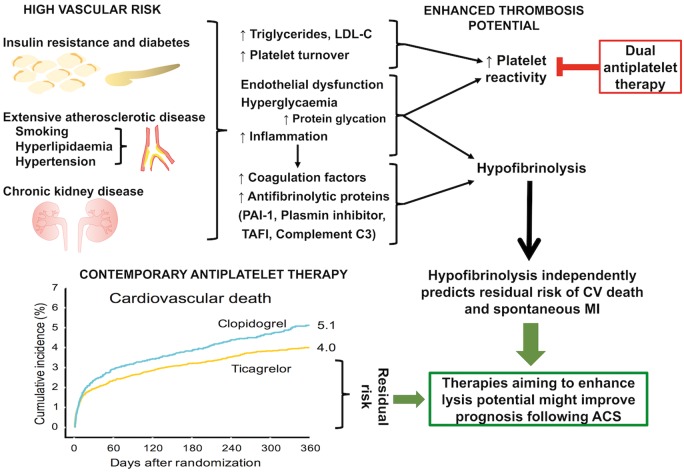

## Introduction

Adverse events, including cardiovascular (CV) death, remain common following acute coronary syndrome (ACS). Intensive antithrombotic therapies, including potent P2Y_12_ inhibitors and the addition of low-dose anticoagulant therapy (rivaroxaban), have all resulted in improved outcomes but increased the risk of major bleeding events.^1–3^

There is marked overlap between risk factors for ischaemic and bleeding events.^4^^,^^5^ Consequently, tailoring therapy to achieve the ‘sweet spot’ of mitigating ischaemia whilst maintaining effective haemostasis is an ongoing challenge and readily available biomarkers to aid the decision process are lacking. Spontaneous major bleeding events are associated with a similar prognosis to ischaemic events^6^ and, in patients undergoing percutaneous coronary intervention (PCI), major bleeding events independently predict major CV events.^7^

Following ACS, treatment includes aspirin and a P2Y_12_ inhibitor.^8^^,^^9^ Although contemporary therapy effectively targets platelets,^10^^,^^11^ around 20% of patients suffer recurrent events within 12 months.^5^ This dual antiplatelet treatment strategy largely spares the protein arm of coagulation, which leads to fibrin formation.

Patients with thrombotic conditions demonstrate unfavourable fibrin clot structure.^12–14^ High-risk conditions, such as diabetes mellitus (DM), chronic kidney disease (CKD), and peripheral artery disease (PAD), have all shown associations with compact fibrin clots and resistance to fibrinolysis.^15–17^ Factors contributing to this phenotype can include levels of clotting and lysis proteins, disease-specific post-translational changes to fibrinogen, and genetic determinants.^18^^,^^19^ Assessment of fibrin clot offers a functional evaluation of the impact of all these factors on the protein arm of coagulation.

The majority of previous studies in this area used a cross-sectional retrospective design and large-scale longitudinal studies are lacking. We, therefore, aimed to study fibrin clot properties in plasma samples collected from ACS patients at hospital discharge and explore the relationship between those characteristics and subsequent clinical outcomes.

## Methods

### Study population and patient samples

The PLATelet inhibition and patient Outcomes (PLATO) trial was an international multi-centre, double-blind, randomized controlled trial of ticagrelor compared with clopidogrel in 18 624 moderate- to high-risk ACS patients. The study design and results have previously been published.^1^^,^^20^ Baseline patient characteristics, including medical and medication history, were recorded at baseline. Study visits were performed at 1, 3, 6, 9, and 12 months. In the PLATO biomarker sub-study,^21^ a subset of 4354 patients provided blood at hospital discharge, which was used for this analysis in order to avoid the effects of anticoagulant therapy used as part of the initial ACS management. Plasma was obtained from citrate-anticoagulated venous blood samples and stored initially at –20°C prior to transfer to Uppsala Clinical Research Centre for storage at –80°C. All study patients provided written informed consent according to a protocol approved by local research ethics committees at participating centres. For our analyses, frozen plasma samples were transferred to the University of Sheffield and stored at –80°C until analysis.

### Fibrin clot assessment

Human thrombin was obtained from Merck Biosciences, recombinant tissue plasminogen activator from Technoclone, calcium chloride dehydrate and Tris from Fisher Scientific, and sodium chloride from Sigma Aldrich. High-throughput turbidimetric analysis was performed in flat-bottomed, polystyrene 96-well plates (Greiner) using a dedicated Multiskan FC (Thermo Scientific) plate reader. Permeation buffer solution (100 mM sodium chloride, 50 mM Tris, and pH 7.4) was used for dilution. Twenty five μL aliquots of plasma (in duplicates) were mixed with 75 μL lysis mix and clots were formed by adding 50 μL activation mix (tissue plasminogen activator 83 ng/mL, calcium chloride 7.5 mM, and thrombin 0.03 U/mL; final concentrations). After shaking for 2 s, plates were read at 340 nm every 12 s at 37°C until lysis in all samples was achieved. Quality control samples were included in all plates. This method has previously been validated.^12^^,^^22–24^ Studied variables included lysis time (time taken for turbidity to drop by 50% from maximum as a measure of lysis potential) and maximum turbidity (turbidity refers to the scattering of light as a measure of fibrin clot density). The co-efficient of variation was 8.3% for lysis time and 3.8% for maximum turbidity. All analyses were performed blinded to clinical outcome and clinical characteristics.

### Other biochemical analyses

Plasma samples obtained at randomization were used to determine other biomarker levels, as previously reported.^21^^,^^25–27^ Briefly, N-terminal pro B-type natriuretic peptide (NT-proBNP), high-sensitivity troponin T, cystatin C, C-reactive protein (CRP), and growth differentiation factor-15 (GDF-15) were measured using sandwich immunoassays. Differential blood count was determined on EDTA-anticoagulated blood samples at randomization.

### Statistical methods

Biomarker levels were natural log-transformed before analysis. Baseline patient characteristics, medical history, and biomarkers were compared across quartile groups of each of the fibrin variables. Continuous data are presented as medians and interquartile ranges and compared using Kruskal–Wallis tests. Categorical data are presented as numbers and percentages and compared using χ^2^ tests. The primary outcome of interest was the composite of CV death and spontaneous myocardial infarction (MI). Secondary outcomes were CV death alone, spontaneous MI alone, stroke, all-cause mortality, definite or probable stent thrombosis according to Academic Research Consortium criteria, PLATO-defined major bleeding, and PLATO-defined bleeding unrelated to coronary artery bypass graft surgery (CABG) (see Supplementary material online for bleeding definitions). Kaplan–Meier curves were derived to compare event rates across the four quartile groups of each of the fibrin clot variables. Cox-proportional hazards models were used to estimate hazard ratios (HRs) and 95% confidence intervals (CIs). Hazard ratios are expressed per 50% increase in fibrin variable level when assessed as continuous variables or compared to the lowest quartile of the fibrin variable when assessed as categorical variables. Two models were used for adjustment. Model 1 included randomized treatment, age, gender, body mass index, smoking history, hypertension, dyslipidaemia, DM, CKD, ST-elevation ACS and previous MI, congestive heart failure (CHF), revascularisation, ischaemic stroke, or PAD.^25–27^ Model 2 included all variables in Model 1 (excluding CKD) and the following inflammatory and prognostic biomarkers: CRP, white blood cell count, cystatin C, NT-proBNP, troponin T, and GDF-15. The assumptions of proportional hazards were assessed visually by calculating Schoenfeld residuals. To assess the prognostic value of fibrin clot properties, Harrell’s C-index was estimated and compared to a clinical predictive model (Model 1) without the addition of fibrin clot variables using likelihood ratio tests. The efficacy and safety of ticagrelor compared with clopidogrel according to fibrin clot properties was assessed using a Cox proportional hazards model that included randomized treatment, continuous fibrin variable level using restricted cubic splines, and randomized treatment by fibrin variable interaction. The effect of fibrin clot properties on clinical outcome in relation to presentation was also assessed using a Cox proportional hazards model that included presentation, continuous fibrin variable level using restricted cubic splines, and presentation-by-fibrin-variable interaction. *P*-values <0.05 from two-tailed tests were considered statistically significant. Due to the exploratory nature of this study, *P*-values were not adjusted for multiple testing. All statistical analyses were performed at Uppsala Clinical Research Centre using R statistics software (Version 3.3.2; R Foundation for Statistical Computing, Vienna, Austria).

## Results

### Relationships between fibrin clot properties, clinical characteristics, and biomarkers

A total of 4354 patients were included in this study. *Table 1* and Supplementary material online, *Table S1* summarise the clinical characteristics and biomarkers across the quartile groups of both lysis time and maximum turbidity.

**Table 1:** Baseline clinical characteristics and biomarkers across lysis time quartile groups

Variables	Lysis time (s) quartile group				*P*-value
	Q1 (<564; *n* = 1098)	Q2 (564–696; *n* = 1108)	Q3 (696–888; *n* = 1066)	Q4 (>888; *n* = 1082)	
Demographics and medical history					
Age (years)	63 (55–72)	61 (54–70)	61 (53–70)	61 (53–70)	<0.001
Female	242 (22.0)	318 (28.7)	271 (25.4)	442 (40.9)	<0.001
Body mass index (kg/m^2^)	27.0 (24.5–29.7)	27.3 (24.9–30.1)	27.8 (25.3–30.8)	28.6 (25.6–31.8)	<0.001
Current smoker	392 (35.7)	450 (40.6)	404 (37.9)	349 (32.3)	<0.001
Hypertension	673 (61.3)	717 (64.7)	711 (66.7)	764 (70.6)	<0.001
Hyperlipidaemia	453 (41.3)	462 (41.7)	456 (42.8)	469 (43.3)	0.744
Diabetes mellitus	206 (18.8)	221 (19.9)	242 (22.7)	305 (28.2)	<0.001
Previous MI	216 (19.7)	215 (19.4)	214 (20.1)	201 (18.6)	0.843
Previous CHF	53 (4.8)	59 (5.3)	58 (5.4)	79 (7.3)	0.068
Previous stroke	34 (3.1)	42 (3.8)	35 (3.3)	40 (3.7)	0.783
PAD	62 (5.6)	68 (6.1)	63 (5.9)	80 (7.4)	0.345
CKD	29 (2.6)	42 (3.8)	27 (2.5)	49 (4.5)	0.028
Type of ACS					
STE-ACS	504 (45.9)	522 (47.1)	510 (47.8)	486 (44.9)	0.536
Biomarkers					
Troponin T (ng/L)	129 (35–453)	151 (37–511)	177 (46–582)	210 (47–755)	<0.001
NT-proBNP (pmol/L)	387 (119–992)	386 (129–1088)	389 (131–1033)	469 (139–1433)	0.009
Cystatin C (mg/L)	0.80 (0.65–0.97)	0.80 (0.66–0.96)	0.81 (0.66–0.97)	0.86 (0.69–1.05)	<0.001
GDF-15 (ng/L)	1503 (1095–2058)	1446 (1108–2092)	1509 (1182–2064)	1584 (1150–2254)	0.003
CRP (mg/L)	2.4 (1.1–5.8)	3.1 (1.4–7.6)	3.9 (1.8–9.8)	5.2 (2.2–13.5)	<0.001
White cell count (×10^9^/L)	8.7 (6.9–10.9)	9.1 (7.3–11.4)	9.7 (7.8–11.9)	9.9 (7.8–12.5)	<0.001
Haemoglobin (g/L)	141 (131–151)	142 (132–151)	142 (132–152)	141 (130–151)	0.144
Haematocrit (L/L)	0.41 (0.39–0.44)	0.42 (0.39–0.44)	0.42 (0.39–0.45)	0.42 (0.38–0.44)	0.265

Values are medians (IQRs) for continuous data and *n* (%) for categorical data. *P*-values calculated using χ^2^ test (categorical variables) or Kruskal–Wallis test (continuous variables).

CHF, congestive heart failure; CKD, chronic kidney disease; CRP, C-reactive protein; GDF, growth differentiation factor; MI, myocardial infarction; NT-proBNP, N-terminal pro b-type natriuretic peptide; PAD, peripheral artery disease; STE-ACS, ST-elevation acute coronary syndrome.

The prevalence of DM, hypertension, CKD, and female sex significantly increased with increasing lysis time quartile group. Although the differences were not significant, the highest quartile group of lysis time appeared to have the highest prevalence of PAD and CHF.

Patients in the shortest lysis time quartile group tended to be older but the absolute difference in mean age was small. Strong associations were also observed between lysis time and other biomarkers: with increasing quartile group, the levels of troponin T, NT-proBNP, CRP, and white blood cell count increased. Growth differentiation factor-15 and cystatin-C levels were also highest in the highest quartile group.

Similar to lysis time, the prevalence of DM was highest in the highest quartile group of maximum turbidity. There was a higher percentage of female patients in the lowest maximum turbidity quartile group. The relationship between maximum turbidity and the other biomarkers was also pronounced (see Supplementary material online, *Table S1*).

Neither lysis time nor maximum turbidity appeared to be affected by haemoglobin or haematocrit.

Maximum turbidity and lysis time had a modest correlation (Spearman correlation co-efficient 0.375, *P* < 0.001).

### Fibrin clot properties and clinical outcome

During follow-up, 125 (2.9%) patients had CV death, 183 (4.2%) patients had spontaneous MI, and 41 (0.94%) patients had stroke. There were 145 all-cause deaths (3.3%). Amongst those treated with PCI (4335), 38 (0.88%) had definite or probable stent thrombosis. PLATO-defined major bleeding occurred in 256 (5.9%) patients with 96 (2.2%) patients having non-CABG-related major bleeding.

**Figure 1:**
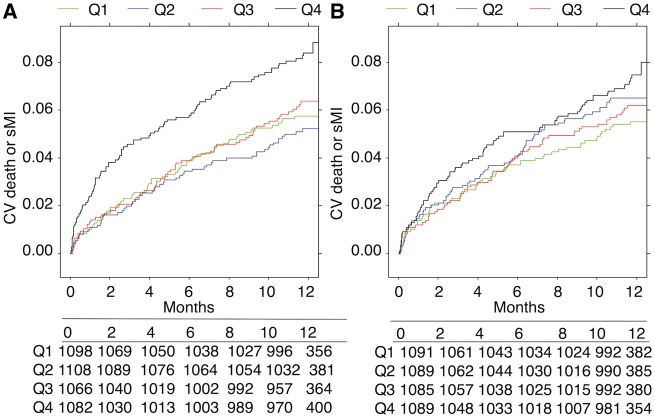
Relationship between fibrin clot parameters and 1-year cumulative event rate of cardiovascular death or spontaneous myocardial infarction. The Kaplan–Meier curves for cumulative event rate of the combined outcome of cardiovascular death or spontaneous myocardial infarction per quartile group of lysis time (*A*) and maximum turbidity (*B*).

**Figure 2:**
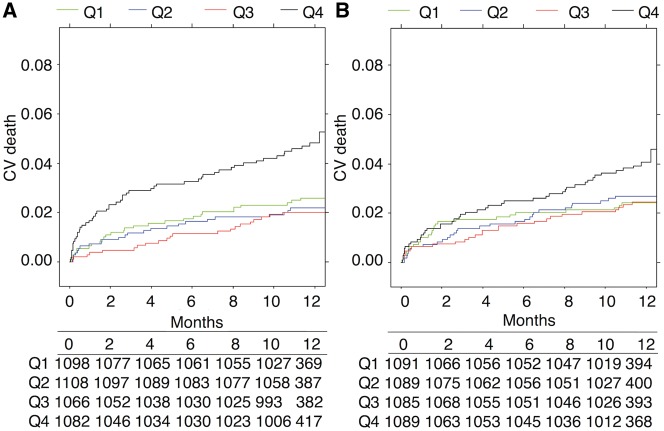
Relationship between fibrin clot parameters and 1-year cumulative event rate of cardiovascular death. The Kaplan–Meier curves for 1-year cumulative event rate of cardiovascular death per quartile group of lysis time (*A*) and maximum turbidity (*B*).

Rates of the composite outcome of CV death and spontaneous MI were higher in the highest quartile groups of both lysis time and maximum turbidity compared with rates in the lowest quartile groups (*Figure 1*). This was primarily driven by higher rates of CV death in the highest quartile groups (*Figure 2*). After adjustment for CV risk factors (Model 1), the highest quartile group of lysis time was associated with increased risk of CV death/spontaneous MI (HR 1.48, 95% CI 1.06–2.06; *P* = 0.027) and CV death alone (HR 1.92, 95% CI 1.19–3.1; *P* < 0.001). As a continuous variable, each 50% increase in lysis time was associated with increased risk of CV death/spontaneous MI (HR 1.17, 95% CI 1.05–1.31; *P* = 0.006) and CV death alone (HR 1.36, 95% CI 1.17–1.59; *P* < 0.001). This association remained significant for lysis time after adjustment for inflammatory and prognostic biomarkers (see Supplementary material online, *Table S2*). Similarly, each 50% increase in maximum turbidity was associated with CV death alone (HR 1.24, 95% CI 1.03–1.50; *P* = 0.024) but this association was no longer significant after adjustment for inflammatory and prognostic biomarkers (see Supplementary material online, *Table S3*). Findings for all-cause mortality were similar to those for CV death (see Supplementary material online, *Tables S2* and *S3*).

There was no clear association with rates of stent thrombosis and stroke but event rates were low (data not shown). Neither lysis time nor maximum turbidity was able to predict major bleeding events (see Supplementary material online, *Tables S2* and *S3*). Further characterisation of bleeding events is provided in the *Supplementary material online, Tables S6 to S8*.

**Figure 3:**
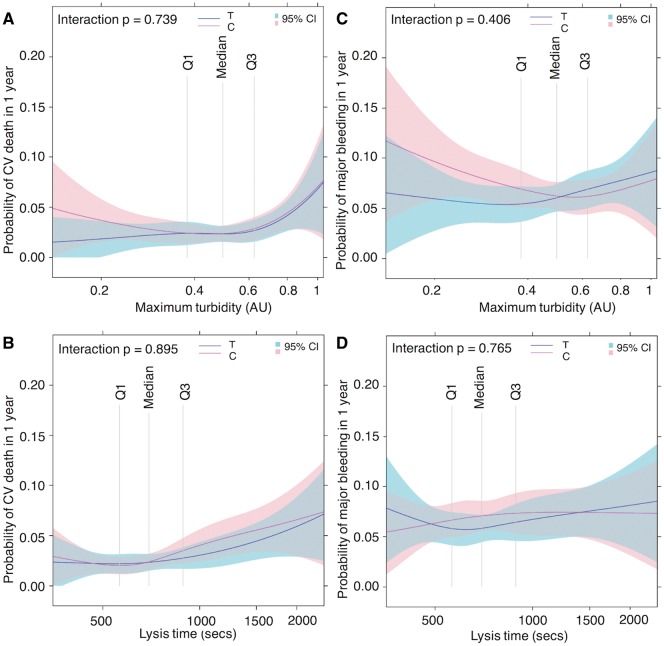
Relationship between fibrin clot parameters and 1-year rates of cardiovascular death or major bleeding according to randomized treatment group. One-year rates of cardiovascular death (*A* and *B*) or major bleeding (*C* and *D*) in relation to maximum turbidity (*A* and *C*) or lysis time (*B* and *D*), transformed using restricted cubic splines, according to randomized treatment with clopidogrel (C, pink lines) or ticagrelor (T, blue lines). Shaded areas represent 95% confidence intervals. Vertical lines indicate quartiles.

There was no significant impact of fibrin clot properties on the CV mortality reduction with ticagrelor compared with clopidogrel (interaction *P* > 0.7) (*Figure 3*). There was also no significant impact of fibrin clot properties on the association between randomized treatment and major bleeding (*Figure 3*). Similarly, the association between fibrin clot properties and CV death was present irrespective of subtype of ACS presentation (all interaction *P* > 0.05). A subset of patients received low-molecular weight heparin on either the day of sampling or the day before (see Supplementary material online, *Tables S4* and *S5*). This treatment did not affect the prognostic value of fibrin clot parameters (all interaction *P* > 0.25).

### Incremental prognostic value of lysis time

Model performance to predict the composite outcome of CV death/sMI significantly improved when lysis time was added to a clinical predictive model: C-index 0.67 (0.637–0.703) for Model 1 + lysis time vs. 0.665 (0.631–0.698) for Model 1 only, *P* = 0.007. Prediction of CV death also significantly improved: C-index 0.7 (0.649–0.75) for Model 1 + lysis time vs. 0.69 (0.642–0.741) for Model 1 only, *P* < 0.001.

## Discussion

We have shown, in a large population of ACS patients treated with contemporary therapies and followed up for up to 1 year, that fibrin clot properties independently predict the risk of spontaneous MI and CV death following initial in-hospital management. Importantly, lysis time predicted worse outcome after adjusting for other established or new prognostic biomarkers, thus indicating the potential for a further biomarker that provides insight into prognosis following ACS. The association between lysis time and the levels of the established biomarkers also raises the possibility that variability in fibrin clot properties might contribute to the association of these other biomarkers with thrombotic events (*Take home figure*). Given that antithrombotic therapy is mainly centred around antiplatelet agents following the acute phase, our data support the hypothesis that at least a subgroup of patients might benefit from additional therapy that aims at improving lysis potential. For example, further work could explore whether anticoagulant therapy, in combination with a platelet P2Y_12_ receptor antagonist, offers an advantage over dual antiplatelet therapy in those with adverse fibrin clot properties. Other novel therapies that specifically target proteins implicated in impaired lysis, such as complement C3 or plasmin inhibitor, is another approach that might have less impact on haemostasis, particularly if the aim was to normalise lysis potential.^18^

**Take home Figure:**
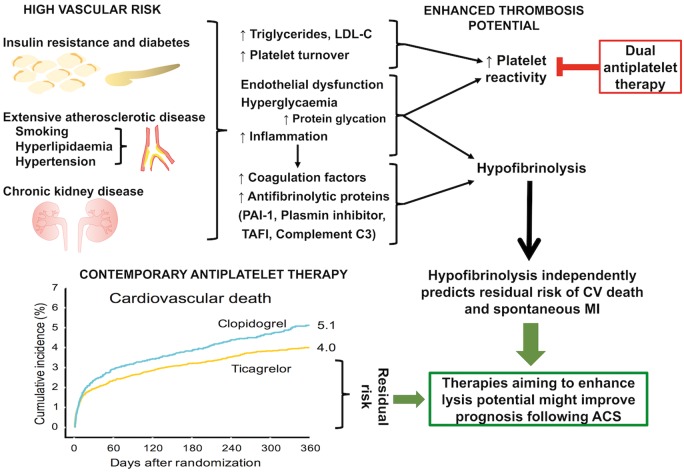
High-risk conditions are associated with increased thrombotic potential through a variety of mechanisms. Dual antiplatelet therapy successfully targets platelet reactivity. However, hypofibrinolysis remains unaffected and constitutes a potential therapeutic target. LDL-C, low-density lipoprotein cholesterol; PAI-1, plasminogen activation inhibitor-1; TAFI, thrombin activatable fibrinolysis inhibitor.

Studying fibrin clot properties is attractive for many reasons. First, fibrinogen conversion and cross-linking of fibrin fibres to form a stable network is a key step in the formation of an obstructive vascular thrombus. Second, thrombotic occlusion of coronary arteries could represent a failure of the protective endogenous thrombolytic mechanisms to lyse clots before they become occlusive. Third, this assay provides a functional and simple assessment of the complex interactions between different clotting/lysis factors and other plasma proteins and takes into account both quantitative and qualitative changes in coagulation factors that may affect fibrinolytic efficiency.^18^^,^^28^ Fourth, this is a relatively cheap, easy and reproducible test to perform and, importantly, fibrin clots that resist lysis might give us mechanistic insights into recurrent events.

Previous studies, using different assays in whole blood, have shown a positive association between prolonged lysis and CV death.^29^^,^^30^ Another study using thromboelastography in plasma showed similar results.^31^ However, these were small studies with limited event rates and assessment of clotting and lysis in whole blood cannot reliably differentiate between the cellular and protein components of thrombus formation.

The relationships between adverse fibrin clot dynamics and some clinical characteristics demonstrated in our work are consistent with evidence obtained from smaller observational, cross-sectional studies. For example, DM, CKD, and PAD, all high-risk conditions for cardiac ischaemia, have shown associations with adverse fibrin clot characteristics.^15–17^

Available biomarkers have added little incremental prognostic value beyond a clinical predictive model and therefore their clinical use has been limited in secondary prevention of CV disease.^32^ Although the added prognostic value of lysis time is also modest, identifying patients with adverse fibrin clot properties might help direct therapy beyond the current approach of antiplatelet therapy in these patients.

Evidence suggests that inflammation results in prothrombotic states.^33^^,^^34^ The strong correlations between fibrin clot properties and inflammatory markers support previous work demonstrating that inflammation leads to prothrombotic changes in fibrin clot dynamics and illustrates one mechanism for the higher risk of atherothrombotic events in patients with higher levels of inflammatory markers.^35^^,^^36^

The relationship between maximum turbidity and worse outcomes lost significance after adjusting for other biomarkers. Fibrinogen levels have a greater effect on maximum turbidity compared with lysis time^24^^,^^37^ (see also Supplementary material online, *Figure S5*) and fibrinogen levels go hand-in-hand with inflammatory markers, particularly CRP.^38^

Interestingly, fibrin clot density and resistance to lysis increased with increasing levels of NT-proBNP and troponin T. The exact molecular mechanism for this association is difficult to ascertain. However, higher troponin T and NT-proBNP reflect larger infarcts and these are associated with a greater inflammatory response, which might account for some of the prothrombotic changes. NT-proBNP has been shown to add prognostic value regardless of the degree of necrosis after ACS.^39^ Our findings, therefore, point to an additional mechanism, beyond the increased risks of death from heart failure and arrhythmia associated with left ventricular systolic dysfunction, whereby NT-proBNP is associated with worse outcome, as a consequence of more dense fibrin clots that resist lysis leading to increased risk of atherothrombosis.

Turbidimetric analysis of fibrin clots requires trained laboratory personnel, and therefore, is not suitable as a bedside test. Similar to other clotting assays, results might be influenced by high-level anticoagulant therapy and significant liver conditions, which make results difficult to interpret in those scenarios. A limitation to this study is that it only provides a ‘snapshot’ assessment of fibrin clot characteristics at hospital discharge (median 6 days). It is established that internal fibrinolytic activity has a circadian rhythm largely driven by variations in plasminogen activator inhibitor—1 activity.^40^ Unfortunately sampling times are not available in our database but samples were collected during office working hours. The clear relationship with DM and biomarker levels, which were all measured at baseline, reassure us that the influence of circadian variation is likely to be marginal. Future analyses will seek to assess the stability of this phenotype over time and how the relationship with clinical outcome could change in a stable patient cohort.

## Conclusions

Despite strong relationships with clinical risk factors, particularly DM, and inflammatory and other prognostic biomarkers, the resistance of fibrin clots to lysis independently predicts CV death following ACS. These findings suggest that novel therapies targeting fibrin clot properties might be a new avenue for improving clinical outcomes in patients with ACS.

## Supplementary material

Supplementary material is available at *European Heart Journal* online.

## Supplementary Material

Supplementary Figure 1

Supplementary Tables
