# Supplementary figures and images for: Fibrin clot properties independently predict adverse clinical outcome following acute coronary syndrome: a PLATO substudy
Source: Eur Heart J. 2018 Jan 29;39(13):1078–85. doi: 10.1093/eurheartj/ehy013 (PMC6019045; doi:10.1093/eurheartj/ehy013)

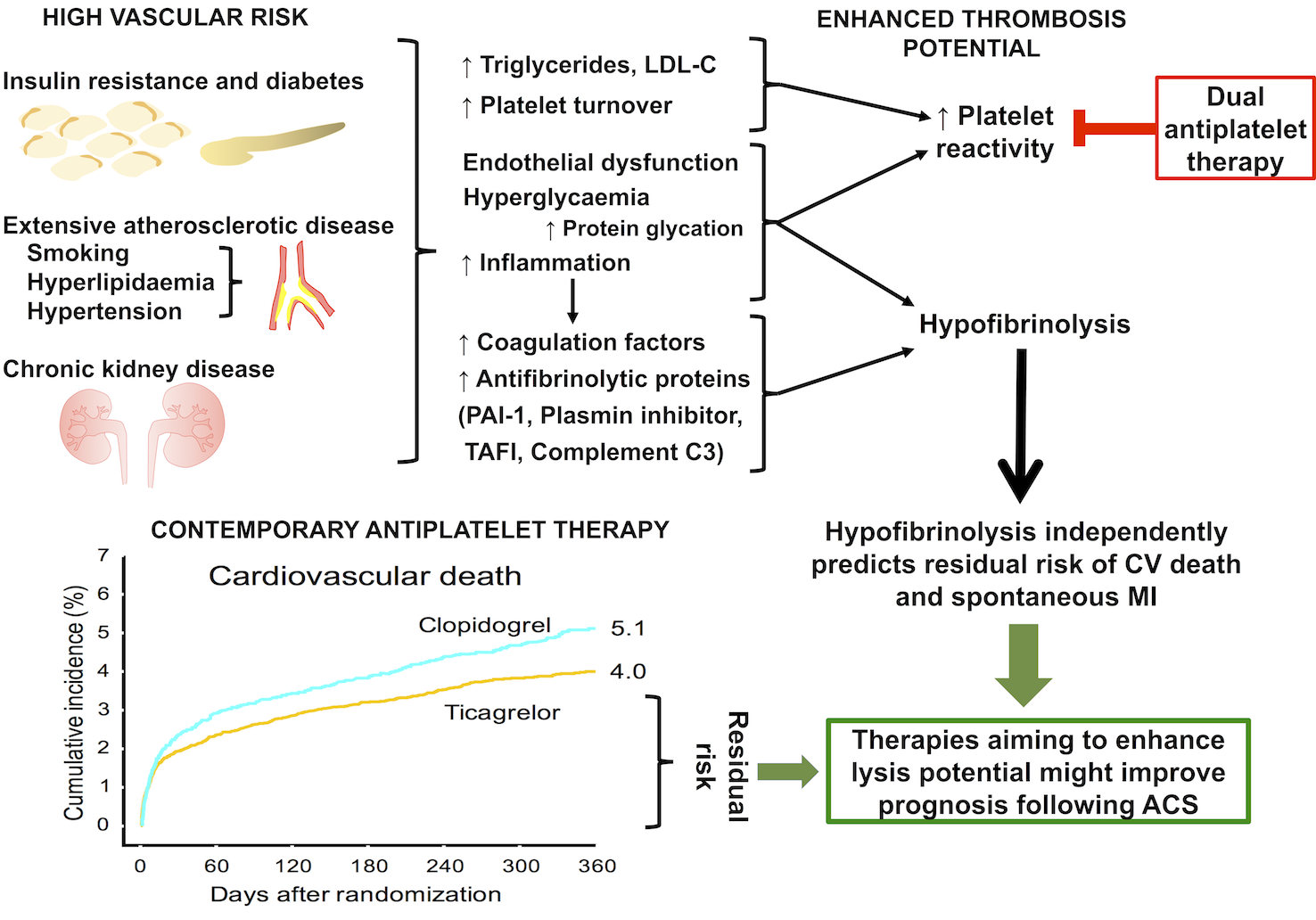

Supplement: Supplementary Figure 1 [file ehy013_supp_figure.png]
