# Supplementary material for: Fibrin clot properties independently predict adverse clinical outcome following acute coronary syndrome: a PLATO substudy
Source: Eur Heart J. 2018 Jan 29;39(13):1078–85. doi: 10.1093/eurheartj/ehy013 (PMC6019045; doi:10.1093/eurheartj/ehy013)
Supplement: Supplementary Tables [file ehy013_supp_results.pdf]

**Supplementary table 1: Baseline clinical characteristics and biomarkers across maximum turbidity quartile groups**

| Variables | Maximum turbidity (au) quartile groups | | | | P value |
|---|---|---|---|---|---|
| | Q1 (≤ 0.38)<br>n = 1091 | Q2 (0.38-0.5)<br>n = 1089 | Q3 (0.5-0.62)<br>n = 1085 | Q4 (>0.62)<br>n = 1089 | |
| **Demographics and medical history** | | | | | |
| Age (years) | 62 (54-71) | 61 (53-70) | 61 (54-70) | 62 (54-70) | 0.272 |
| Female | 366 (33.5%) | 334 (30.7%) | 327 (30.1%) | 246 (22.6%) | < 0.001 |
| BMI (kg/m$^2$) | 27 (24.6-29.9) | 27.8 (25.2-30.8) | 27.8 (25.3-30.8) | 28 (25.2-31.1) | < 0.001 |
| Current smoker | 358 (32.8%) | 405 (37.2%) | 409 (37.7%) | 423 (38.8%) | 0.02 |
| Hypertension | 747 (68.5%) | 690 (63.4%) | 715 (65.9%) | 713 (65.5%) | 0.094 |
| Hyperlipidaemia | 496 (45.5%) | 492 (45.2%) | 432 (39.8%) | 420 (38.6%) | < 0.001 |
| Diabetes mellitus | 222 (20.3%) | 225 (20.7%) | 256 (23.6%) | 271 (24.9%) | 0.026 |
| Previous MI | 259 (23.7%) | 214 (19.7%) | 194 (17.9%) | 179 (16.4%) | < 0.001 |
| Previous CHF | 90 (8.2%) | 54 (5.0%) | 48 (4.4%) | 57 (5.2%) | < 0.001 |
| Previous stroke | 43 (3.9%) | 41 (3.8%) | 29 (2.7%) | 38 (3.5%) | 0.381 |
| PAD | 79 (7.2%) | 63 (5.8%) | 55 (5.1%) | 76 (7%) | 0.123 |
| CKD | 28 (2.6%) | 37 (3.4%) | 33 (3.0%) | 49 (4.5%) | 0.079 |
| **Type of ACS** | | | | | |
| STE-ACS | 392 (35.9%) | 467 (42.9%) | 553 (51%) | 610 (56%) | < 0001 |
| **Biomarkers** | | | | | |
| Troponin T (ng/L) | 91 (21 – 301) | 129 (31 – 410) | 163 (44 – 494) | 375 (97 – 1161) | < 0.001 |
| NT-proBNP (pmol/L) | 335 (124 – 867) | 332 (112 – 904) | 390 (118 – 989) | 662 (191 – 1774) | < 0.001 |
| Cystatin C (mg/L) | 0.82 (0.66–0.99) | 0.79 (0.65 –0.96) | 0.81 (0.66 –0.98) | 0.84 (0.68 –1.02) | 0.002 |
| GDF-15 (ng/L) | 1447 (1084-2007) | 1417 (1065-1993) | 1504 (1144-2076) | 1692 (1243-2414) | < 0.001 |
| CRP (mg/L) | 2.4 (1.1 – 5.2) | 2.7 (1.4 – 6.0) | 3.9 (1.7 – 8.6) | 6.8 (2.7 – 22) | < 0.001 |
| WCC (*10$^9$/L) | 8.2 (6.6 – 10.3) | 8.9 (7.2 – 11.1) | 9.7 (7.7 – 11.8) | 10.7 (8.7 – 13.1) | < 0.001 |
| Haemoglobin (g/L) | 141 (130-150) | 142 (132-151) | 141 (131-150) | 142 (132-152) | 0.12 |
| Haematocrit (L/L) | 0.42 (0.38-0.44) | 0.42 (0.39-0.44) | 0.41 (0.39-0.44) | 0.42 (0.39-0.45) | 0.05 |

Values are medians (IQRs) for continuous data and n (%) for categorical data. AU: Arbitrary units; other abbreviations as per table 1. P values calculated using Chi-square test (categorical variables) or Kruskal-Wallis test (continuous variables)

**Supplementary table 2: Associations between lysis time and clinical outcomes**

| Event | Model | Continuous lysis time | P value | Lysis time quartile groups (HR compared to the lowest quartile group) | | | P value |
|---|---|---|---|---|---|---|---|
| | | HR per 50% increase | | Q2 (564-696 secs) | Q3 (696-888 secs) | Q4 (>888 secs) | |
| CV death and sMI | Model 1 | 1.17 (1.05-1.31) | 0.006* | 0.92 (0.64-1.32) | 1.09 (0.77-1.55) | 1.48 (1.06-2.06) | 0.027* |
| | Model 2 | 1.15 (1.01-1.30) | 0.032* | 0.88 (0.60-1.31) | 1.09 (0.75-1.60) | 1.40 (0.97-2.03) | 0.088 |
| CV death | Model 1 | 1.36 (1.17-1.59) | <0.001* | 0.87 (0.50-1.51) | 0.79 (0.45-1.39) | 1.92 (1.19-3.10) | <0.001* |
| | Model 2 | 1.20 (1.01-1.42) | 0.042* | 0.82 (0.46-1.47) | 0.79 (0.43-1.44) | 1.44 (0.85-2.45) | 0.08 |
| All-cause death | Model 1 | 1.39 (1.20-1.61) | <0.001* | 1.04 (0.62-1.77) | 1.24 (0.74-2.06) | 2.24 (1.40-3.57) | 0.001 |
| | Model 2 | 1.21 (1.03-1.42) | 0.021* | 0.95 (0.54-1.67) | 1.08 (0.62-1.88) | 1.61 (0.96-2.7) | 0.131 |
| sMI | Model 1 | 1.08 (0.94-1.25) | 0.287 | 0.83 (0.53-1.28) | 1.14 (0.76-1.72) | 1.17 (0.77-1.77) | 0.36 |
| | Model 2 | 1.12 (0.96-1.31) | 0.15 | 0.80 (0.50-1.29) | 1.13 (0.73-1.77) | 1.24 (0.79-1.96) | 0.29 |
| Major bleeding | Model 1 | 1.09 (0.96-1.23) | 0.18 | 1.03 (0.72-1.47) | 1.07 (0.75-1.53) | 1.27 (0.89-1.80) | 0.56 |
| | Model 2 | 1.07 (0.94-1.23) | 0.31 | 1.04 (0.72-1.51) | 1.04 (0.71-1.52) | 1.20 (0.81-1.76) | 0.81 |

Estimates are HRs (95% CI). Model 1 included adjustments for age, gender, BMI, diabetes, dyslipidaemia, hypertension, chronic kidney disease (CKD), smoking, type of ACS, randomized treatment and previous myocardial infarction, revascularization, peripheral artery disease, congestive heart failure or cerebrovascular disease. Model 2 included adjustments as per model 1 (excluding CKD) and CRP, leukocyte count, cystatin C, NT-proBNP, high-sensitivity troponin T and GDF 15. CV: cardiovascular; sMI: spontaneous myocardial infarction. * indicates a statistically significant result.

**Supplementary table 3: Associations between maximum turbidity and clinical outcomes**

| Event | Model | Continuous maximum turbidity | P value | Maximum turbidity quartile groups (HR compared to the lowest quartile group) | | | P value |
|---|---|---|---|---|---|---|---|
| | | HR per 50% increase | | Q2 (0.38-0.5 au) | Q3 (0.5-0.62 au) | Q4 (>0.62 au) | |
| **CV death and sMI** | Model 1 | 1.13 (0.99-1.27) | 0.06 | 1.3 (0.92-1.84) | 1.21 (0.85-1.72) | 1.44 (1.02-2.03) | 0.2 |
| | Model 2 | 1.01 (0.88-1.16) | 0.87 | 1.25 (0.85-1.83) | 1.03 (0.69-1.54) | 1.14 (0.76-1.70) | 0.64 |
| **CV death** | Model 1 | 1.24 (1.03-1.50) | 0.024* | 1.21 (0.71-2.07) | 1.04 (0.60-1.81) | 1.77 (1.07-2.9) | 0.08 |
| | Model 2 | 0.96 (0.79–1.16) | 0.65 | 1.04 (0.59-1.84) | 0.80 (0.44-1.44) | 0.88 (0.50-1.57) | 0.8 |
| **All-cause death** | Model 1 | 1.22 (1.03-1.46) | 0.024* | 1.17 (0.71-1.92) | 1.10 ( 0.67-1.83) | 1.71 (1.07-2.72) | 0.09 |
| | Model 2 | 0.96 (0.80-1.15) | 0.639 | 1 (0.58-1.71) | 0.85 (0.49-1.48) | 0.93 (0.54-1.59) | 0.93 |
| **sMI** | Model 1 | 1.08 (0.93-1.25) | 0.332 | 1.25 (0.82-1.9) | 1.26 (0.83-1.93) | 1.28 (0.83-1.95) | 0.63 |
| | Model 2 | 1.06 (0.89-1.27) | 0.483 | 1.27 (0.80-2.03) | 1.18 (0.73-1.91) | 1.35 (0.82-2.23) | 0.64 |
| **Major bleeding** | Model 1 | 0.99 (0.87-1.12) | 0.89 | 0.87 (0.61-1.24) | 0.96 (0.68-1.35) | 1 (0.71-1.41) | 0.86 |
| | Model 2 | 0.93 (0.81-1.07) | 0.324 | 0.8 (0.55-1.17) | 0.94 (0.65-1.36) | 0.85 (0.57-1.27) | 0.68 |

Estimates are HRs (95% CI). AU: arbitrary units; other abbreviations as per table 3. Models 1 and 2 with adjustments as per supplementary table 1. * indicates a statistically significant result.

**Supplementary table 4: In-patient treatment per quartile group of lysis time**

| Variables | Lysis time (secs) quartile group | | | | P value |
|---|---|---|---|---|---|
| | Q1 (<564)<br>n = 1098 | Q2 (564-696)<br>n = 1108 | Q3 (696-888)<br>n = 1066 | Q4 (>888)<br>n = 1082 | |
| **Invasive treatment** | 769 (70%) | 793 (71.6%) | 784 (73.5%) | 742 (68.6%) | 0.069 |
| **Aspirin** | 1081 (99%) | 1094 (99%) | 1055 (99%) | 1057(98%) | 0.085 |
| **LMWH** | 608 (55%) | 608 (55%) | 593 (56%) | 573 (53%) | 0.591 |
| **LMWH on day before sampling** | 414 (37.9%) | 403 (36.6%) | 422 (39.9%) | 398 (37.2%) | 0.429 |
| **LMWH on day of sampling** | 311 (28.5%) | 303 (27.5%) | 321 (30.3%) | 297 (27.8%) | 0.471 |
| **Fondaparinux** | 16 (1.5%) | 17 (1.5%) | 17 (1.6%) | 24 (2.2%) | 0.498 |
| **Beta blockers** | 959 (87.3%) | 969 (87.5%) | 929 (87.1%) | 938 (86.7%) | 0.954 |
| **ACE-I or ARB** | 954 (87%) | 978 (88%) | 925 (87%) | 944 (87%) | 0.711 |
| **Statins** | 1051 (96%) | 1060 (96%) | 1004 (94%) | 998 (92%) | < 0.001 |

P values calculated using Chi-square test.

**Supplementary table 5: In-patient treatment per quartile group of maximum turbidity**

| Variables | Maximum turbidity quartile | | | | P value |
|---|---|---|---|---|---|
| | **Q1 (≤ 0.38)**<br>**n = 1091** | **Q2 (0.38-0.5)**<br>**n = 1089** | **Q3 (0.5-0.62)**<br>**n = 1085** | **Q4 (>0.62)**<br>**n = 1089** | |
| **Invasive treatment** | 637 (58.4%) | 765 (70.2%) | 820 (75.6%) | 866 (79.5%) | <0.001 |
| **Aspirin** | 1077 (99%) | 1074 (99%) | 1065 (98%) | 1071 (98%) | 0.702 |
| **LMWH** | 613 (56%) | 613 (56%) | 584 (54%) | 572 (53%) | 0.212 |
| **LMWH on day before sampling** | 436 (40.3%) | 401 (37.1%) | 408 (37.9%) | 392 (36.3%) | 0.26 |
| **LMWH on day of sampling** | 351 (32.4%) | 303 (28%) | 285 (26.5%) | 293 (27.2%) | 0.01 |
| **Fondaparinux** | 15 (1.4%) | 19 (1.7%) | 19 (1.8%) | 21 (1.9%) | 0.787 |
| **Beta blockers** | 923 (85%) | 957 (88%) | 965 (89%) | 950 (87%) | 0.019 |
| **ACE-I or ARB** | 935 (86%) | 946 (87%) | 961 (89%) | 959 (88%) | 0.183 |
| **Statins** | 1010 (93%) | 1027 (94%) | 1038 (96%) | 1038 (95%) | 0.007 |

P values calculated using Chi-square test.

**The relationship between fibrin clot properties and clinical outcomes according to in-patient treatment (invasive vs. conservative)**

The effect of fibrin clot properties on clinical outcome in relation to treatment (invasive vs. conservative) was assessed using a Cox proportional hazards model that included treatment strategy, continuous fibrin variable level using restricted cubic splines and treatment strategy by fibrin variable interaction. Results are shown in supplementary figure below.

**Supplementary figure 1: Relationship between fibrin clot parameters and 1-year rates of CV death according to treatment strategy**

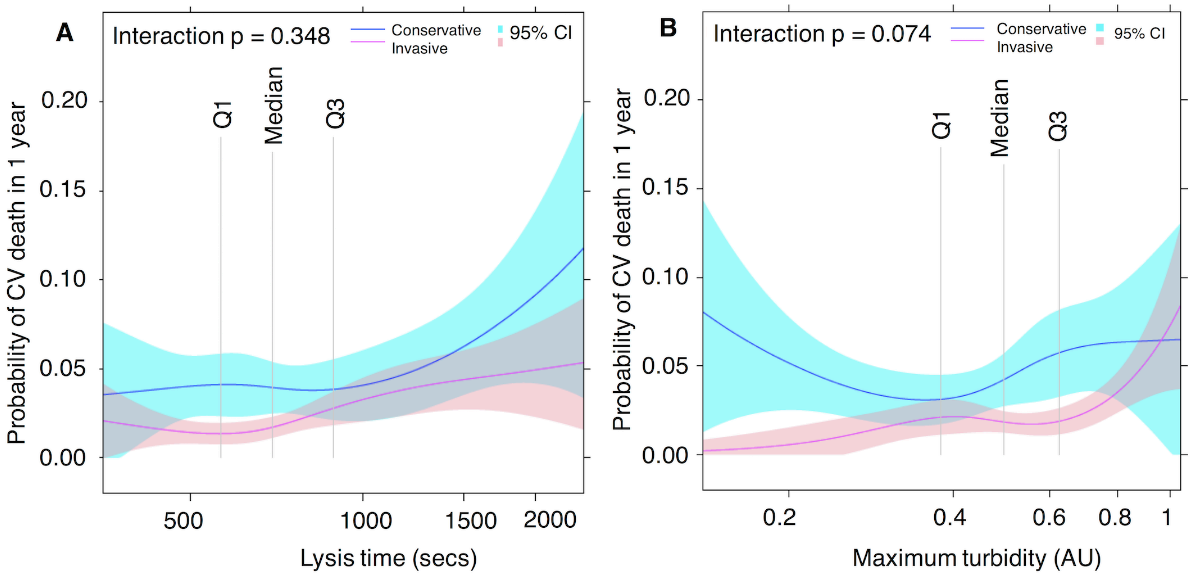


**1-year rates of CV death in relation to lysis time (Panel A) and maximum turbidity (Panel B), transformed using restricted cubic splines, according to treatment strategy with invasive treatment (pink lines) or conservative treatment (blue lines). Shaded areas represent 95% confidence intervals. Vertical lines indicate quartiles.**

**Fibrin clot variables and all-cause mortality**

145 patients died during the follow-up period. After adjustment for clinical risk factors (model 1) every 50% increase in lysis time was associated with increased mortality risk (HR 1.39; 95% CI 1.2-1.61; P < 0.001) (Supplementary figure 2). This association remained significant after adjusting for model 2 variables. The association between maximum turbidity and all-cause mortality was less pronounced and lost significance after adjustment for model 2 variables.

**Supplementary figure 2: Relationship between lysis time (A), maximum turbidity (B) and all-cause mortality at 1 year**

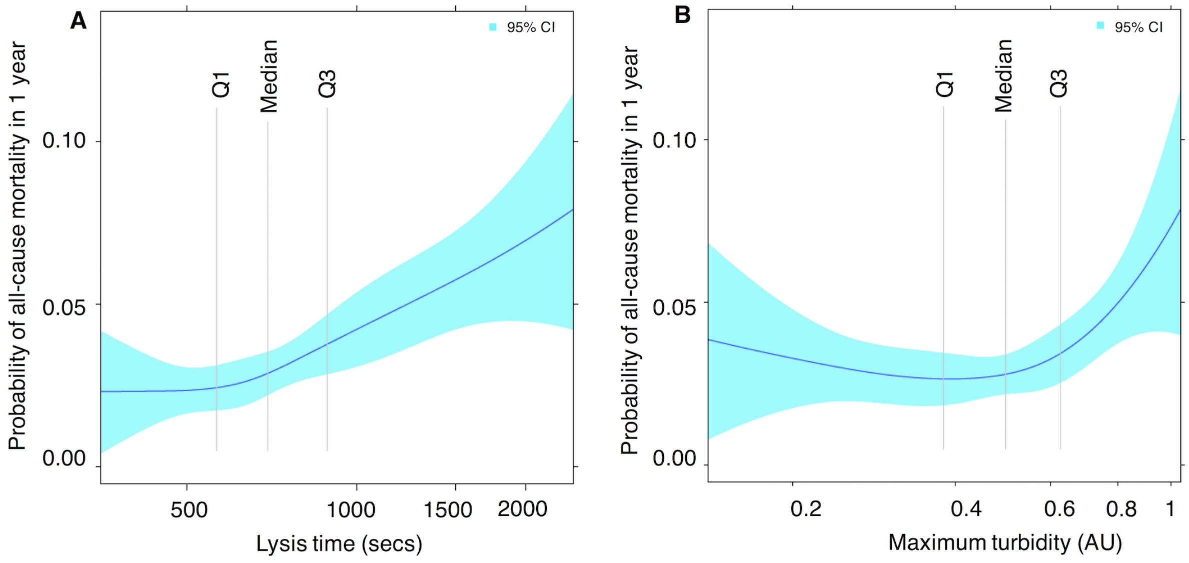


**1-year rates of all-cause mortality in relation to lysis time (A) and maximum turbidity (B) transformed using restricted cubic splines. Shaded areas represent 95% confidence intervals. Vertical lines indicate quartiles.**

**PLATO-defined bleeding definitions [1]:**

- **Major life-threatening:** Fatal, intracranial, intrapericardial with cardiac tamponade, hypovolemic shock or severe hypotension due to bleeding and requiring pressors or surgery, a decline in the haemoglobin level of 5 g/dL or more, requiring transfusion of at least 4 units of red cells.
- **Other major bleeding:** Leading to clinically significant disability (eg. Intraocular bleeding with permanent vision loss), associated with a drop in haemoglobin of at least 3 g/dL and less than 5 g/dL, requiring transfusion of 2-3 units of red cells.
- **Minor bleeding:** Any bleeding requiring medical intervention but not meeting the above-mentioned criteria.

**Supplementary table 6: Bleeding events per quartile groups of lysis time**

| **Type of bleeding** | **All** | **Lysis time (secs) quartile group** | | | |
|---|---|---|---|---|---|
| | | **Q1 (<564)** n = 1098 | **Q2 (564-696)** n = 1108 | **Q3 (696-888)** n = 1066 | **Q4 (>888)** n = 1082 |
| **Fatal** | 4 (0.09%) | 1 (0.09%) | 0 (0.00%) | 2 (0.19%) | 1 (0.09%) |
| **Major CABG-related** | 165 (3.82%) | 41 (3.77%) | 47 (4.28%) | 38 (3.59%) | 39 (3.64%) |
| **Major Non-CABG related** | 96 (2.22%) | 22 (2.02%) | 17 (1.55%) | 28 (2.64%) | 29 (2.71%) |
| **Other major non-CABG related** | 11 (0.26%) | 5 (0.46%) | 0 (0.00%) | 1 (0.09%) | 5 (0.47%) |
| **Life-threatening/fatal non-CABG related** | 16 (0.37%) | 3 (0.28%) | 3 (0.27%) | 6 (0.57%) | 4 (0.37%) |
| **Intracranial** | 5 (0.12 %) | 1 (0.09%) | 1 (0.09%) | 2 (0.19%) | 1 (0.09%) |
| **Minor** | 101 (2.34%) | 21 (1.93%) | 28 (2.55%) | 23 (2.17%) | 29 (2.71%) |
| **Major or minor** | 344 (7.97%) | 79 (7.26%) | 89 (8.11%) | 82 (7.74%) | 94 (8.77%) |
| **Major, minor or minimal** | 381 (8.83%) | 90 (8.27%) | 96 (8.75%) | 93 (8.78%) | 102 (9.52%) |

**Supplementary table 7: Bleeding events per quartile groups of maximum turbidity**

| Type of bleeding | All | Maximum turbidity (AU) quartile group | | | |
|---|---|---|---|---|---|
| | | Q1 (≤ 0.38) n = 1091 | Q2 (0.38-0.5) n = 1089 | Q3 (0.5-0.62) n = 1085 | Q4 (>0.62) n = 1089 |
| **Fatal** | 4 (0.09 %) | 0 (0.00 %) | 1 (0.09%) | 1 (0.09%) | 2 (0.19%) |
| **Major CABG-related** | 165 (3.82%) | 45 (4.17%) | 39 (3.61%) | 41 (3.80%) | 40 (3.71%) |
| **Major non-CABG related** | 96 (2.22%) | 24 (2.22%) | 18 (1.67%) | 24 (2.22%) | 30 (2.79%) |
| **Other major non-CABG related** | 11 (0.26%) | 4 (0.37%) | 2 (0.19%) | 1 (0.09%) | 4 (0.37%) |
| **Life-threatening/fatal non-CABG related** | 16 (0.37%) | 4 (0.37%) | 4 (0.37%) | 3 (0.28%) | 5 (0.46%) |
| **Intracranial** | 5 (0.12%) | 0 (0.00%) | 2 (0.18%) | 1 (0.09%) | 2 (0.18%) |
| **Minor** | 101 (2.34%) | 28 (2.59%) | 23 (2.13%) | 23 (2.13%) | 27 (2.51%) |
| **Major or minor** | 344 (7.97%) | 92 (8.52%) | 76 (7.04%) | 84 (7.79%) | 92 (8.54%) |
| **Major, minor or minimal** | 381 (8.83%) | 100(9.26%) | 87 (8.06%) | 94 (8.71%) | 100 (9.29%) |

**Supplementary table 8: Clinical outcome per quartile groups of both lysis time and maximum turbidity combined.**

| Variable | All | <888 secs lysis time + <0.63 AU turbidity | <888 secs lysis time + >0.62 AU turbidity | >888 secs lysis time + <0.62AU turbidity | >888 secs lysis time + >0.62 AU turbidity |
|---|---|---|---|---|---|
| **CV death/sMI** | 275 (6.316 %) | 148 (5.640 %) | 37 (5.710 %) | 47 (7.332 %) | 43 (9.751 %) |
| **CV death** | 125 (2.871 %) | 59 (2.248 %) | 14 (2.160 %) | 22 (3.432 %) | 30 (6.803 %) |
| **sMI** | 183 (4.203 %) | 105 (4.002 %) | 26 (4.012 %) | 30 (4.680 %) | 22 (4.989 %) |
| **Stroke** | 41 (0.942 %) | 22 (0.838 %) | 6 (0.926 %) | 7 (1.092 %) | 6 (1.361 %) |
| **Major bleeding** | 256 (5.931 %) | 147 (5.652 %) | 39 (6.065 %) | 41 (6.426 %) | 29 (6.682 %) |
| **Non-CABG related major bleeding** | 96 (2.224 %) | 51 (1.961 %) | 16 (2.488 %) | 15 (2.351 %) | 14 (3.226 %) |

**Interaction analysis between fibrin clot parameters and LMWH treatment on either day of sampling or day before**

The effect of fibrin clot properties on clinical outcome in relation to LMWH treatment on day of sampling or the day before was assessed using a Cox proportional hazards model that included LMWH treatment, continuous fibrin variable level using restricted cubic splines and LMWH by fibrin variable interaction. Results are shown in supplementary figures 3 and 4 below.

**Supplementary figure 3: Relationship between fibrin clot parameters and 1-year rates of CV death according to treatment with LMWH received the day before sampling**

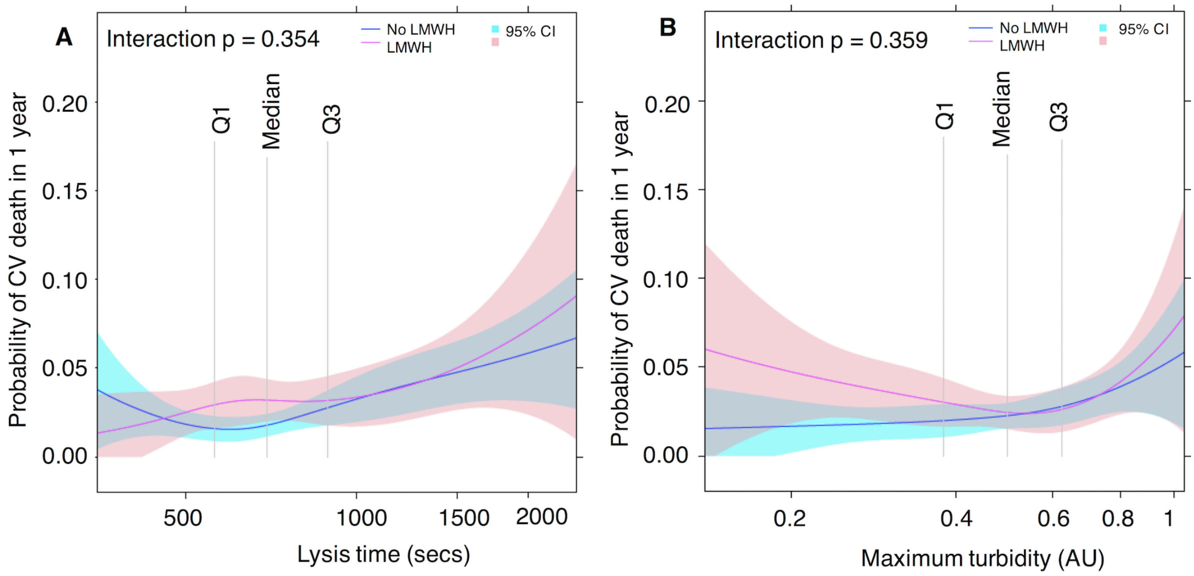


**1-year rates of CV death in relation to lysis time (Panel A) and maximum turbidity (Panel B), transformed using restricted cubic splines, according to LMWH treatment with LMWH the day before sampling (pink lines) or no LMWH (blue lines). Shaded areas represent 95% confidence intervals. Vertical lines indicate quartiles.**

**Supplementary figure 4: Relationship between fibrin clot parameters and 1-year rates of CV death according to treatment with LMWH received the day of sampling**

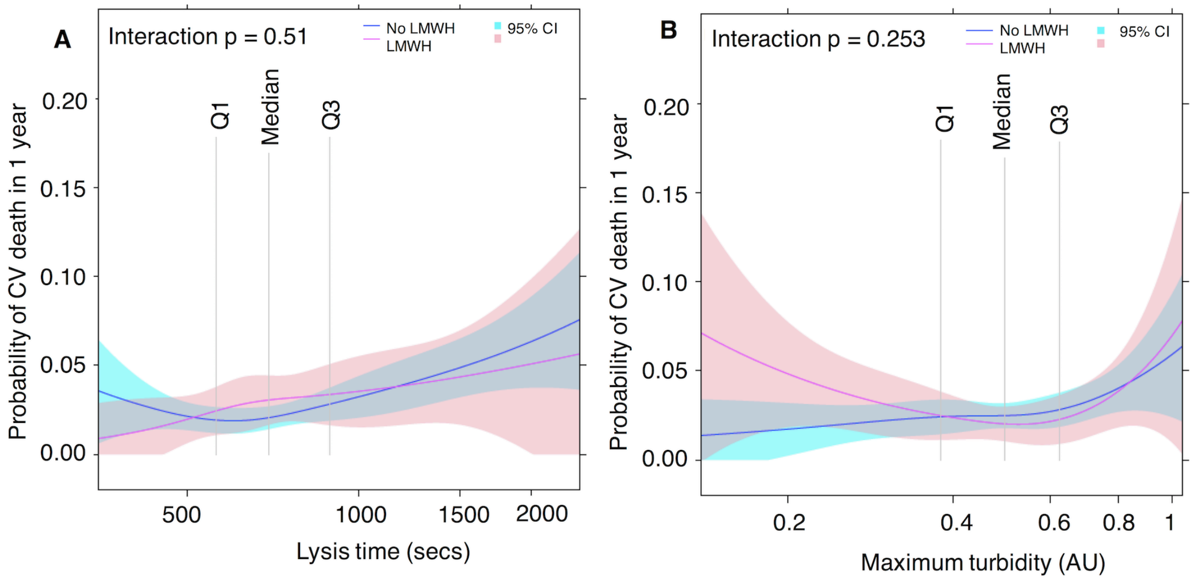


**1-year rates of CV death in relation to lysis time (Panel A) and maximum turbidity (Panel B), transformed using restricted cubic splines, according to LMWH treatment with LMWH received on sampling day (pink lines) or no LMWH (blue lines). Shaded areas represent 95% confidence intervals. Vertical lines indicate quartiles.**

**Correlations between lysis time, maximum turbidity and fibrinogen levels**

Using plasma samples collected at day 7 and stored from a local ACS cohort in Sheffield, United Kingdom,[2, 3] we performed pilot studies to assess the relationship between fibrin clot parameters and fibrinogen levels.

Fibrin clots were studied using the same turbidimetric assay. Fibrinogen levels were determined using Clauss methodology. Significant correlations were present between fibrinogen levels and maximum turbidity but there was no clear relationship present between lysis time and fibrinogen levels (Supplementary figure 5). These results indicate that fibrinogen levels play a more pronounced role in fibrin clot density compared to lysis potential.

**Supplementary figure 5: Correlations between fibrinogen levels and maximum turbidity (A) and lysis time (B)**

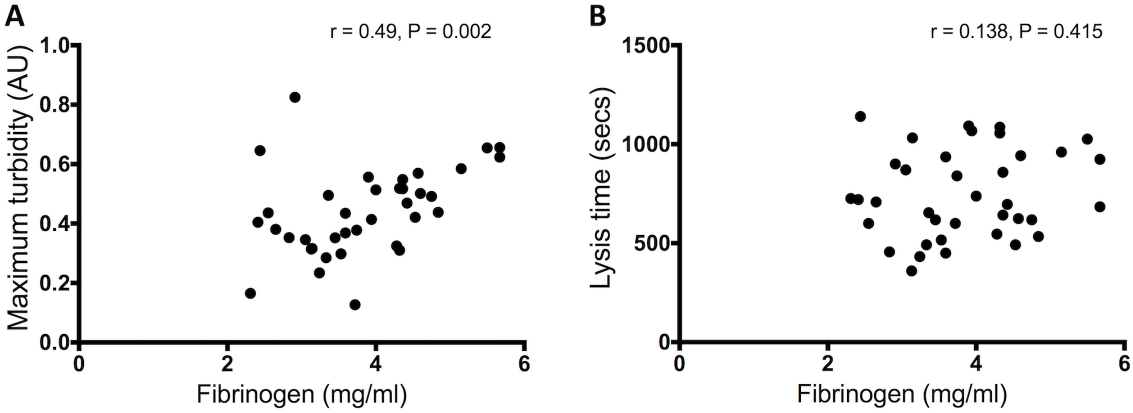


n=37, r value refers to Spearman's correlation co-efficient.

## REFERENCES


1. Wallentin L, Becker RC, Budaj A, Cannon CP, Emanuelsson H, Held C, Horrow J, Husted S, James S, Katus H, Mahaffey KW, Scirica BM, Skene A, Steg PG, Storey RF, Harrington RA, Investigators P, Freij A, Thorsen M. Ticagrelor versus clopidogrel in patients with acute coronary syndromes. N Engl J Med 2009;361:1045-57.

2. Joshi RR, Hossain R, Morton AC, Ecob R, Judge HM, Wales C, Walker JV, Karunakaran A, Storey RF. Evolving pattern of platelet P2Y12 inhibition in patients with acute coronary syndromes. Platelets 2014;25:416-22.

3. Kaudewitz D, Skroblin P, Bender LH, Barwari T, Willeit P, Pechlaner R, Sunderland NP, Willeit K, Morton AC, Armstrong PC, Chan MV, Lu R, Yin X, Gracio F, Dudek K, Langley SR, Zampetaki A, de Rinaldis E, Ye S, Warner TD, Saxena A, Kiechl S, Storey RF, Mayr M. Association of MicroRNAs and YRNAs With Platelet Function. Circ Res 2016;118:420-432.
